# Healthcare Facility Preference among Households in Addis Health and Demographic Surveillance Site (ADDIS-HDSS), Addis Ababa, Ethiopia

**DOI:** 10.4314/ejhs.v34i2.5S

**Published:** 2024-12

**Authors:** Yoseph Yemane Berhane, Workagegnhu Tarekegn, Dagmawit Tewahido, Semira Abdelmenan, Nebiyou Fasil, Hanna Yemane Berhane, Sitota Tsegaye, Dongqing Wang, Uttara Partap, Wafaie Fawzi, Meaza Demissie, Alemayehu Worku, Yemane Berhane

**Affiliations:** 1 Department of Epidemiology and Biostatistics, Addis Continental Institute of Public Health; 2 Department of Nutrition and Behavioral Science, Addis Continental Institute of Public Health; 3 Department of Global Health and Health Policy, Addis Continental Institute of Public Health; 4 Department of Global and Community Health, College of Public Health, George Mason University, Fairfax, Virginia, United States of America; 5 Department of Global Health and Population, Harvard T.H. Chan School of Public Health, Harvard University, Boston, Massachusetts, United States of America

**Keywords:** Addis HDSS, Healthcare preference, Universal Health Coverage, Private versus Public healthcare facilities, Healthcare seeking, LMICs

## Abstract

**Background:**

Understanding healthcare facility preferences—public versus private—is essential for formulating policies that promote universal health coverage (UHC). Various sociodemographic factors influence these preferences. This study examines healthcare facility preferences within the ADDIS-HDSS population in Addis Ababa, Ethiopia.

**Methods:**

This analysis is based on the ADDIS-HDSS baseline census conducted in Yeka sub-city, Addis Ababa. We focused on respondents' preferred healthcare facility when household members are ill, and the reasons behind their choice. Multivariable logistic regression was used to analyze the factors influencing preferences.

**Results:**

Most households (82.81%) preferred public healthcare facilities, citing affordability as the primary reason (59.31%). The remaining 17.19% preferred private healthcare, mainly due to perceived quality of care (43.06%) and timeliness (37.04%). Larger family size was associated with a reduced likelihood of preferring private facilities (AOR = 0.579, 95% CI: 0.522–0.642), while higher education (AOR = 2.573, 95% CI: 2.194–3.017) and wealthier households (AOR = 16.925, 95% CI: 14.705–19.481) were more likely to prefer private care.

**Conclusion:**

The majority of households prefer public healthcare facilities, with affordability, quality, and timeliness as key factors. To achieve UHC in low-income countries, improving service quality and timeliness in public healthcare is critical.

## Introduction

In low- and middle-income countries (LMICs), equitable access to healthcare remains a major challenge. A critical aspect of this challenge is understanding the population's preferences for healthcare facilities. The balance between public and private sectors is especially important in the context of efforts to achieve universal health coverage (UHC).

In many LMICs, the public sector plays a central role in achieving UHC due to its broader reach, particularly in rural areas, and its more affordable services compared to the private sector, which is often concentrated in urban centers. Despite the rising costs of healthcare, many people in LMICs continue to rely on the public sector due to subsidized costs and waivers. However, the private sector also plays an important role in offering specialized services, making it essential to understand how these two sectors interact and the factors influencing healthcare preferences.

Healthcare facility preferences are shaped by various factors, including accessibility, affordability, and quality of care. Sociodemographic and economic characteristics also play a significant role. In Ethiopia, a low-income country, strides have been made toward achieving UHC, although disparities between urban and rural areas persist. The public sector predominantly addresses preventive healthcare needs, while the private sector is more focused on curative services.

The dynamic nature of healthcare preferences underscores the need for ongoing research to inform policymaking. Understanding the factors that shape healthcare preferences—such as economic status, education, and healthcare system organization—is crucial for designing effective health policies and ensuring sustainable healthcare services. This study investigates the healthcare facility preferences of residents in an urban setting in Addis Ababa, Ethiopia.

## Methods

**Study setting**: This study was conducted as part of the Addis Health and Demographic Surveillance System (ADDIS-HDSS) from December 2022 to January 2023, in the Yeka sub-city of Addis Ababa. Yeka is one of the largest sub-cities in Addis Ababa, with two public hospitals, ten public health centers, and 84 private health facilities. Most of these facilities are easily accessible by public transportation. Public facilities offer subsidized or free services for preventive and curative care, particularly for maternal and child health, and chronic diseases like diabetes, tuberculosis, and HIV/AIDS. Private facilities, on the other hand, are market-driven and depend on the providers' expertise and the profitability of services.

**Study design and population**: The study used baseline census data from the ADDIS-HDSS, which included 30,533 households. The primary study population consisted of adult household members, primarily household heads. Households with no adult members available during the survey were excluded (n = 627, 2.05%).

**Data collection**: Data collection tools were adapted from standardized INDEPTH Network tools and similar studies conducted in Ethiopia. Tools were available in both English and Amharic. Data was collected in-person at household residences, with up to three attempts made to reach each household. Data collectors used Open Data Kit (ODK) software for data entry, and extensive training was provided on study protocols, ethics, and survey procedures.

**Data analysis**: Data was analyzed using STATA 14 software. Family size was categorized into small (1-2 members), medium (3-4 members), and large (5 or more members). Wealth was stratified into quintiles, from the poorest to the wealthiest households. The outcome variable was the healthcare facility preference—either public or private—based on the household's choice when members are ill. Reasons for the preference were also recorded.

Descriptive statistics were used to summarize categorical variables. Binary and multivariable logistic regression models assessed the association between healthcare facility preference and explanatory variables. Factors with a p-value less than 0.20 in bivariate analysis were included in the final model. Adjusted odds ratios (AOR) and 95% confidence intervals (CI) were reported, with statistical significance set at p < 0.05.

**Ethical considerations**: Ethical clearance was obtained from the Addis Continental Institute of Public Health Ethical Review Committee (ACIPH/IRB/003/2022). Participants were fully informed about the study, and written consent was obtained. Confidentiality and anonymity were maintained throughout the study.

## Results

**Sociodemographic characteristics**: A total of 29,906 households participated in the study. The mean age of household heads was 47.7 ± 16.08 years, with 60.7% being male. Around 26.3% of household heads had completed primary education, while 26.7% had attained a college degree. More than half (56.7%) of household heads were married, and 28.7% were unemployed (Table 1).

**Table 1:** Socio-demographic characteristics of the study households in Yeka Sub City, Addis Ababa, Ethiopia 2023

Category (n=29906)	Number	Percent
**Age Group**		
18 to 39	12937	43.26%
40 to 59	10504	35.12%
60 to 79	5382	18.00%
80+	1083	3.62%
***Mean Age = 47.7 (±16.08)***		
**Sex**		
Male	18152	60.70%
Female	11754	39.30%
**Education Level**		
No Formal Education	3674	12.23%
Primary school/Lower	7853	26.26%
Secondary school	8781	29.36%
Vocational/technical	1624	5.43%
College/University	7974	26.66%
**Employment**		
Unemployed	8591	28.73%
Employed	21315	71.27%
**Household Family Size**		
Small family	8888	29.72%
Medium family	13204	44.15%
Large family	7814	26.13%
**Marital Status**		
Single (never married)	4800	16.10%
Married (monogamous)	16899	56.68%
Divorced	2042	6.85%
Widow/widower	5058	16.96%
Separated	1107	3.70%
**Wealth Index**		
Lowest	6504	21.75%
Second	5462	18.26%
Middle	5989	20.03%
Fourth	5987	20.02%
Highest	5964	19.94%

**Figure 1:**
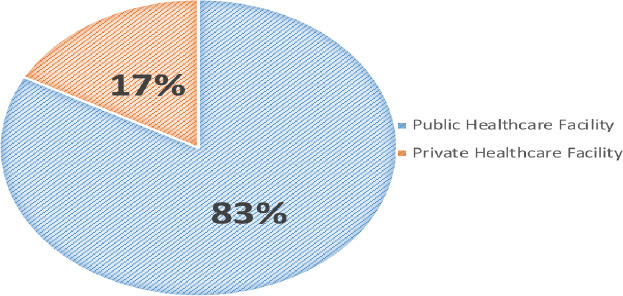
Households preference of healthcare facilities; public versus private

**Healthcare facility preference**: The majority (82.81%) of household heads preferred public healthcare facilities to meet their families' healthcare needs, while 17.19% preferred private facilities (see Figure 1).

**Figure 2:**
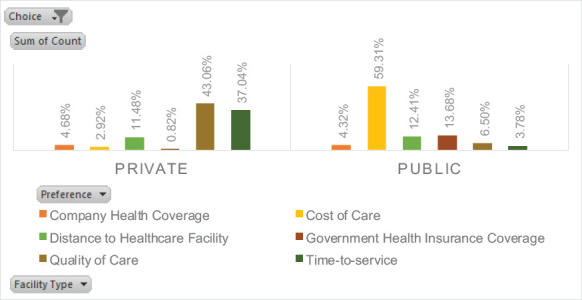
Reasons for healthcare facility preference during illness, Addis Yeka HDSS Site, Addis Ababa, Ethiopia, 202

The primary reason for preferring public healthcare was affordability, cited by 59.31% of households. Other factors influencing the preference for public facilities included the distance to healthcare centers (12.41%) and government health insurance coverage (13.65%) (see Figure 2). Among those who favored private healthcare, the main factors were perceived quality of care (43.06%) and timeliness of service (37.01%) (see Figure 2).

**Factors associated with healthcare facility preferences**: Age did not emerge as a significant predictor of healthcare facility preference. However, several sociodemographic factors and household characteristics influenced preferences.

Households with higher education levels were more likely to prefer private healthcare facilities (AOR = 2.572, 95% CI: 2.194–3.017). Married individuals were less likely to opt for private care compared to singles (AOR = 0.651, 95% CI: 0.582–0.728). Similarly, divorced (AOR = 0.445, 95% CI: 0.479–0.686) and separated individuals (AOR = 0.579, 95% CI: 0.552–0.844) were less likely to choose private care. Households with larger family sizes were less likely to seek private healthcare compared to those with smaller families (AOR = 0.579, 95% CI: 0.522–0.642), while wealthier households were more likely to choose private facilities (AOR = 16.925, 95% CI: 14.705–19.481) (Table 2).

**Table 2:** Factors that influence healthcare facility preference in ADDIS-HDSS

Variable	n	COR(95% CI)	AOR(95% CI)
**Age**	29906	1.005(1.003,1.006)*	0.994(0.991,0.998)*
**Household Head Sex**			
Male	18152	1	1
Female	11754	0.806(0.757,0.858)*	0.975(0.889,1.070)*
**Education Status**			
No Formal Education	3674	1	1
Primary school/lower	7853	1.120(0.968,1.297)	1.114(0.952,1.304)
Secondary School	8781	2.114(1.845,2.420)*	1.523(1.306,1.789)*
Vocational/Technical	1624	2.856(2.403,3.402)*	1.585(1.299,1.933)*
College/University	7974	5.689(4.992,6.483)*	2.573(2.194,3.017)*
**Employment Status**			
Unemployed	8591	1	1
Employed	21315	1.107(1.0342,1.183)*	1.062(0.962,1.172)
**Marital Status**			
Single	4800	1	1
Married(monogamous)	16899	0.758(0.700,0.820)*	0.651(0.582,0.728)*
Divorced	2042	0.445(0.381,0.520)*	0.574(0.479,0.686)*
Widow/widower	5058	0.698(0.630,0.773)*	0.824(0.709,0.956)*
Separated	1107	0.579(0.481,0.696)*	0.683(0.552,0.844)*
**Family size**	29906		
Small	8888	1	1
Medium	13204	0.747(0.696,0.803)*	0.642(0.586,0.704)*
Large	7672	0.978(0.905,1.057)	0.579(0.522,.642)*
**Wealth Index**	29906		
Lowest	6504	1	1
Second	5462	1.422(1.217,1.663)*	1.419(1.211,1.662)*
Middle	5989	2.147(1.861,2.476)*	2.032(1.756,2.351)*
Fourth	5987	4.613(4.046,5.260)*	4.787(4.167,5.500)*
Highest	5964	16.844(14.866,19.085)*	16.925(14.705,19.481)*

## Discussion

This study found that most households preferred public healthcare facilities, driven primarily by cost, quality of care, and timeliness. Educational level, marital status, family size, and household wealth were also significant factors.

The preference for public healthcare aligns with patterns seen across many Sub-Saharan African countries, where cost is a major consideration. Public facilities serve large populations and prioritize access over service quality. While private providers may offer better-qualified staff and perceived higher-quality care, they often charge more, which can be a deterrent for lower-income households.

Public facilities also offer a broader range of services, particularly preventive care, such as vaccination and family planning, which may contribute to the preference for these centers. Additionally, public facilities tend to establish closer relationships with clients, fostering trust.

While fewer households preferred private healthcare, those who did cited the perceived higher quality and shorter waiting times. These findings align with previous research showing that socioeconomic factors like education and wealth strongly influence healthcare utilization patterns. Educated households and those with higher incomes are more likely to afford and seek private care, which offers faster and more specialized services.

The observation that female-headed households were less likely to opt for private healthcare supports previous studies indicating a greater tendency among women to prioritize preventive care. Additionally, the preference for public healthcare among married individuals and those with larger families may reflect concerns over the high out-of-pocket costs associated with private care.

The high preference for public healthcare could place strain on public facilities, leading to longer wait times and potential declines in service quality. This highlights the need for strategies to balance public and private sector involvement in achieving universal health coverage (UHC).

This study provides a comprehensive, community-based understanding of healthcare preferences. However, it did not link preferences to specific illnesses, nor did it fully capture the perspectives of female caregivers, who may have different views from household heads (often male). The study also did not explore whether preferences varied based on child or adult illness.

In conclusion, 80% of households preferred public healthcare facilities, mainly due to the low cost of services. In contrast, private healthcare was favored for its perceived higher quality and shorter wait times. These findings underscore the importance of improving both affordability and service quality in healthcare systems. A significant portion of the population, especially those with low socioeconomic status, large families, and low educational attainment, relies on public facilities. To achieve universal health coverage, improving the quality and timeliness of services in public healthcare facilities, along with optimizing costs in the private sector, is crucial.
